# Healthy Food Environments in Food Pantries: Lessons Learned from a Sodium Reduction Intervention

**DOI:** 10.3390/ijerph182413206

**Published:** 2021-12-15

**Authors:** Emilee L. Quinn, Kate Ortiz, Laura Titzer, Barb Houston-Shimizu, Jessica Jones-Smith

**Affiliations:** 1 Department of Health Systems and Population Health, University of Washington, Seattle, WA 98195, USA; jjoness@uw.edu; 2 Chronic Disease & Injury Prevention Division, Public Health Seattle and King County, Seattle, WA 98104, USA; kate.ortiz@kingcounty.gov; 3 Northwest Harvest, Seattle, WA 98134, USA; laurat@northwestharvest.org; 4 South King County Food Coalition, Des Moines, WA 98198, USA; director@skcfc.org; 5 Department of Epidemiology, University of Washington, Seattle, WA 98195, USA

**Keywords:** sodium, nutrition policy, guidelines, food insecurity, diet, food preferences, environment, evaluation, implementation, fruit and vegetables

## Abstract

In the United States, food pantries increasingly serve as regular food sources for low income households experiencing high rates of chronic disease, including hypertension. Sodium consumption is a modifiable risk factor for hypertension, so pantry customers would benefit from access to low-sodium foods. Pantry customers often experience difficulty acquiring healthy foods, however; little is known about pantry foods’ sodium content specifically. This study assesses the sodium content of pantry foods and lessons learned from an adaptable intervention to support pantries in adopting policies and environmental changes to make healthy, lower-sodium foods appealing and accessible. We conducted sodium assessments of food at 13 food pantries, tracked implementation of intervention strategies, and interviewed 10 pantry directors. More than half of food items in 11 categories met sodium standards for foods to be chosen “often”. Pantry directors reported valuing the intervention approach and implemented six of nine behavioral economics strategies, especially those targeting the visibility and convenience of foods, along with layout changes and expanded customer choice. One pantry adopted an agency-specific nutrition policy and 12 adopted a coalition-level policy. Results can inform intervention efforts to make available healthy options appealing and easy to select while also improving the customer experience in food pantries.

## 1. Introduction

In the United States (US), charitable food agencies, such as food pantries increasingly serve as regular food sources for households living with low income [1]. Populations living with low income and food insecurity experience higher rates of nutrition-related chronic diseases and the diets of pantry customers often do not align with dietary guidelines [2,3,4,5]. Nearly half of adults in the US live with hypertension (high blood pressure), often uncontrolled, which is a risk factor for heart disease and stroke [6]. Adults who are non-Hispanic black or over the age of 65, and those who report low income or food insecurity experience higher rates of hypertension than the general population and are overrepresented among food pantry customers [7,8,9]. Given that sodium consumption is a modifiable risk factor for hypertension, pantry customers would benefit from access to lower-sodium foods [10]. Relatively little is known about sodium levels of pantry foods specifically, but pantry customers—especially those living with chronic diseases—report wanting more healthy foods from pantries [5,11,12,13,14].

A range of policy and environmental strategies have been proposed to promote access to healthy foods in food pantries through changes to the food supply, food environment, and access to education and other services [15]. While nutrition insecurity is an important concern for households living with low income, food pantries often perceive that they have limited control over the food they distribute since much is donated. Additionally, pantry food environments tend to be structured around the need to serve many people with limited space and staff rather than customer experience. Pantries are increasingly interested in offering healthy and culturally-relevant food, however, and research has shown that pantry-based interventions can improve dietary outcomes [16,17]. Still, much remains to be learned about the acceptability and feasibility of applying evidence-based best practices in “real world” pantry settings [18].

A sizeable body of literature highlights the importance of community and consumer food environments on dietary and health outcomes, while also making clear that these relationships are complex, particularly for households living with low incomes [19,20,21]. Households experiencing food insecurity may be less likely to report easy or affordable access to high quality food options even if a retail outlet offering such foods is located nearby [22]. At the same time, research indicates that dietary and health outcomes vary based on where food is acquired; for example, convenience stores and other non-traditional food outlets are associated with the poorer nutritional quality of foods and health outcomes than other store types [23,24]. Charitable food sources are often not included in such comparisons, despite the increased regularity of their use among households living with low income. Other factors of the retail food environment known to influence perceived and actual food access include cost; food option variety and quality; food outlet accessibility, cleanliness, and customer service; and promotions and product placement [22]. Relatively few studies of food pantry environments account for such factors or those unique to pantry settings, such as the distribution process or the extent of food choice. They also typically do not consider how the food pantry environment might interact with psychosocial factors commonly experienced by people seeking food from charitable food sources.These psychosocial factors can include poverty, stigma, stress, language barriers, health conditions, or lack of housing.

In 2016, partners in King County, Washington State in the US began implementing a food pantry intervention aimed at promoting access to healthy, lower-sodium foods through environmental and policy change efforts. The intervention was supported through the United States (US) Centers for Disease Control and Prevention’s Sodium Reduction in Communities Program which aims to increase the availability of lower sodium foods in the food supply at the institutional and community level. This paper describes lessons learned during the first two years of implementation with regard to the sodium content of foods in the food pantries, perceptions of the intervention approach, and environmental and policy development outcomes achieved by the first 13 pantries to participate.

## 2. Materials and Methods

### 2.1. Intervention

The project team developed the Healthy Food Environments (HFE) intervention to support food pantries in making healthy, lower-sodium foods more appealing and accessible through (1) implementing environmental changes informed by behavioral economics and customer choice strategies and (2) developing nutrition-based procurement policies. Project partners included representatives from the local health department, a statewide food bank distributor (which supplies food to food pantries), an extension-based nutrition education program, a university-based public health nutrition research group, and a local food pantry coalition. Twelve food pantries comprising the coalition, plus one affiliated with the food bank, were the first to participate in the project.

Pantries participating in the intervention received a baseline sodium assessment of their pantry’s foods, training and technical assistance, signage materials, and $2000 for supplies and staff time. Training and technical assistance included two 1-hour workshops and consultation on behavioral economics and nutrition policy development. Behavioral economics is the “study of social, cognitive, and emotional factorswhich aims to understand and influence economic decisions or purchasing behaviors” [25] (p. 4). Changes based on behavioral economics have been shown to increase the selection of targeted food items in experimental and real-life settings including food pantries [25,26,27,28,29]. The HFE intervention aimed to promote access to and appeal of healthy, lower-sodium items by encouraging pantries to employ strategies, such as: increasing the visibility or convenience of the items; placement of such items early or repeatedly in the shopping experience; use of signage, recipes, cooking demonstrations, or nutrition information to highlight the items; communicating the normalcy or popularity of such items; and bundling such items with other ingredients to promote selection. The technical assistance team also provided consultation aimed at improving the customer experience and increasing opportunities for customer choice (e.g., allowing customers to select their own food options and use of a “grocery model” food distribution format). Such “choice strategies” are increasingly considered a best practice in pantry settings, but have a relatively limited evidence base [16,30,31,32]. These strategies were added to the intervention after early feedback from pantry participants that the intervention should aim to improve the customer experience while also promoting access to healthy options. Site-specific consultation from the team included two or more visits to observe baseline pantry operations, brainstorming and discussing the opportunities for and application of environmental changes with staff and volunteers, developing a site layout plan for implementing the changes, and ordering and installing supplies. The intervention also provided signage with “Rinse to Reduce” messaging to encourage rinsing canned beans and vegetables to reduce sodium. The intervention was designed to be adaptable so each organization could make changes best-suited to their context [33]. To participate, pantries committed to implementing at least three of the highlighted environmental change strategies, adopting a nutrition policy, and engaging in data collection. Pantries received support for up to 12 months.

### 2.2. Recruitment

The project team advertised the intervention at a coalition meeting and invited pantries to apply. The application assessed pantries’ readiness to participate (e.g., anticipated challenges, capacity). The technical assistance team selected five pantries to participate in the first year based on their readiness and eight to participate in the second year.

### 2.3. Data Collection

#### 2.3.1. Sodium Assessments

Assessing the sodium content of foods in the food pantry setting is difficult due to limitations in pantry inventory systems (e.g., records may quantify pounds of foods received or distributed, but often not types or brands of foods) and the heterogeneous and changing food inventory of most pantries given that many foods are typically donated rather than purchased. We developed and piloted a rapid assessment approach at one pantry, and upon finding it feasible, repeated the process for all pantries participating in the intervention. At the outset of working with each pantry, the HFE team assessed the baseline sodium content of pantry foods for one distribution. The baseline assessments were generally conducted in a three- to four-hour period prior to a pantry’s food distribution when as much food as possible was set out, but no customers were present. In one case, the assessment took place during distribution. The assessments were intended to: (1) provide insight into the sodium content of pantry foods; (2) provide actionable information about the potential for highlighting healthy, lower-sodium foods within pantry settings and (3) establish a baseline for future comparison following any procurement changes instituted via nutrition policies. Policy implementation efforts and follow-up sodium assessments were delayed due to the COVID-19 pandemic.

Data collectors recorded the brand name, key details (e.g., flavor, low-fat, low-sodium), weight or volume, number of servings, calories per serving, and sodium (milligrams) per serving for each unique food item using information from the item’s packaging and nutrition label, and the quantity of each food item. If an item did not have a nutrition label or information was illegible, as much information as possible was recorded. When pantries had more foods out for distribution than could be assessed in the allotted time, foods with nutrition labels were prioritized. Non-processed produce items were not assessed since these contain minimal sodium.

#### 2.3.2. Implementation Process Records

Application forms completed by food pantry directors included questions about pantry staffing, model and frequency of food distribution, food sources, and customer reach prior to participation in the intervention. Some descriptors were also collected from the pantries’ websites. The HFE team documented environmental changes made in each pantry based on site visits and consultations with pantry staff and volunteers in an implementation tracking spreadsheet throughout the intervention. Individual pantries completed a worksheet reporting their planned approach to policy implementation after policy adoption.

#### 2.3.3. Interviews

Two team members conducted semi-structured interviews with directors of ten pantries at the end of the second year to assess the acceptability, feasibility, and early impacts of the intervention. Three pantries were not interviewed due to scheduling conflicts (n = 1) or delays in implementation (n = 2). Interview questions addressed rationales for and perceived benefits of participating, environmental changes made, the policy development process, and the technical assistance provided. Interviews took 30–60 min and were recorded and transcribed. The University of Washington Human Subjects Division determined these activities were exempt from review.

### 2.4. Data Analysis

#### 2.4.1. Sodium Assessments

Data from all sites were entered into one Excel file. Each assessed food item was then assigned to one of 18 food categories based on definitions from the *Healthy Eating Research Nutrition Guidelines for the Charitable Food System (Guidelines)* [34]. The *Guidelines* were first published in 2020 and developed by a national panel of nutrition and charitable food system experts based upon a review of existing guidelines, including the *2015–2020 Dietary Guidelines for Americans*, combined with an understanding of common food sourcing challenges in the charitable food system. Although the *Guidelines* were published after study data were collected, we used them for analysis since they are likely to be widely referenced in the charitable food sector going forward. The *Guidelines* rank 11 food categories into three tiers (choose “often,” “sometimes,” or “rarely”) based on standards for saturated fat, sodium, and added sugar. We assigned all assessed foods to one of the *Guidelines’* categories, or a subcategory for more detailed examination (e.g., protein-canned beans), and excluded categories with 10 or fewer items. We calculated descriptive statistics using IBM SPSS Statistics 19. We then compared the median level of sodium (mg) per serving to the *Guidelines*’ sodium standards for that category and assigned it to one of the *Guidelines’* three ranked tiers. Standards varied by food category and tier, but items generally needed to have 230 mg of sodium or less to be classified as “choose often” except for mixed dishes, which had to have 480 mg of sodium or less per serving. We calculated the percentage of foods from each category in each tier.

#### 2.4.2. Implementation Process Records

Data on food pantry characteristics and use of environmental change strategies were entered into Excel and descriptive statistics (e.g., counts, minimum, maximum, mean) were calculated.

#### 2.4.3. Interviews

One team member developed a codebook based on the interview questions and used Atlas.ti (7.5.18) to code all interview transcripts. The team member summarized themes pertaining to rationales for participating in the intervention, how the intervention supported changes made, challenges experienced, and environmental change and policy outcomes. Key themes were reviewed with the technical assistance team and coalition members and compared to implementation tracking data as a means of data triangulation.

## 3. Results

### 3.1. Pantry Characteristics

All participating food pantries reported purchasing no or relatively small proportions of food, relying instead on donations. In other respects, they were diverse. Participating pantries served between 112 and 4000 customers during service hours ranging from three to 33 hours per week (Table 1). Eleven pantries distributed food via a service line (customers selected or were handed food), three used a full or partial “grocery store” model (open floor format with customer-directed shopping), and three distributed some or all foods in pre-packed bags or boxes. They reported having a median of 2.5 full-time staff, 1.5 part-time staff, and 44 volunteers. Customer languages included English, Spanish, Vietnamese, Russian, Somali, Chinese, Ukrainian, Cambodian, and Arabic.

### 3.2. Sodium Content of Foods

The sodium content of 25,451 food items was assessed. Processed vegetables, mixed dishes (e.g., soup, macaroni and cheese), and canned/dried fruit were assessed in the largest number. Non-dairy alternatives (e.g., plant-based milk) were assessed least, followed by cereal (Table 2). Data were missing for 10% or more of items from eight food categories because the items lacked a nutrition label; these items were typically items donated from grocers’ bakeries or delis or were bulk items from food bank distributors.

**Figure 1:**
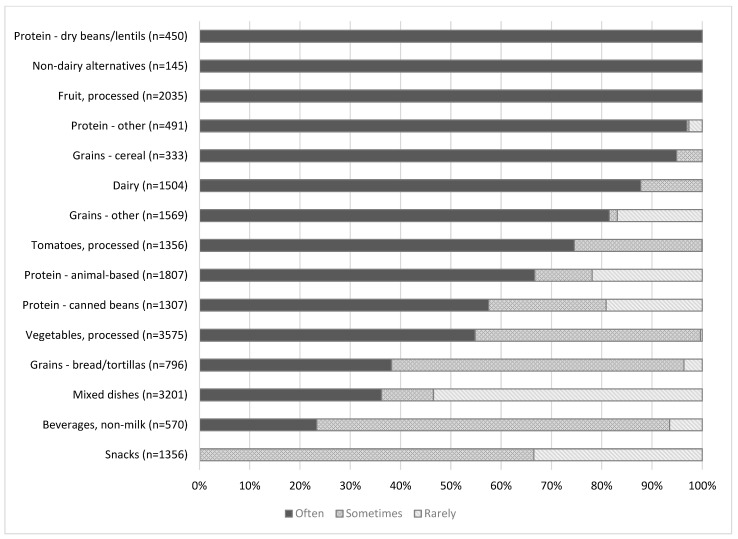
Percent of items categorized as foods to be eaten often, sometimes, or rarely calculated by the authors using sodium standards from the *Healthy Eating Research Nutrition Guidelines for the Charitable Food System (Guidelines)* in 13 food pantries in King County, Washington 2017–2018. The *Guidelines* outline standards for saturated fat, sugar, and in some cases, whole grain ingredients, in addition to sodium for each tier [34]. Here, we only account for the sodium specification. Based on the nutrition standards, snack foods could only be classified as “choose sometimes” or “choose rarely.” Condiments, cooking staples, and desserts are not included because sodium standards are not specified for these foods.

Of the 15 food categories ranked across multiple tiers, the median sodium level per serving of items in 11 categories fell into the “choose often” tier, three into the “choose sometimes” tier, and one into the “choose rarely” tier. All dry bean/lentils, non-dairy alternatives, and fruit items met standards for the “often” tier (Figure 1). More than two-thirds of “other” proteins (e.g., nuts, nut butters, tofu), cereal, dairy, other grains (e.g., pasta, rice), canned tomatoes, and animal/fish-based protein met “choose often” standards; between one-half and two-thirds of canned beans and canned vegetables met “choose often” standards. Less than half of bread, mixed dishes, and beverages met “choose often” standards. The *Guidelines* classifies all processed snacks as items to choose “sometimes” or “rarely.”More than half (54%) of mixed dishes (e.g., items with multiple whole ingredients, such as canned soup and boxed meals) met the criteria for “choose rarely.” Mixed dishes had the highest median sodium level per serving.

### 3.3. Environmental Change Outcomes

Pantries implemented six of nine behavioral economics strategies (Table 3). The most common strategies involved improving the visibility and convenience of healthy and lower-sodium foods so they would be more likely to be selected (*n* = 11). Seven pantries changed their layout to improve customer flow through the pantry (e.g., foods that had been distributed outdoors were brought indoors, areas that resulted in longer waits for customers were addressed) so that customers would not be deterred from or rushed through selecting foods from some displays. Eight pantries offered more opportunities for customer choice or created a pantry experience more like a grocery store. No pantries chose to implement nutrition labeling, normalizing, or bundling strategies.

In interviews, most directors cited specific environmental changes as the primary benefit of participating in HFE. Transitioning to a grocery store model—including customer-selection of foods, customer-direction of shopping pathways, and use of carts—was noted by five directors.

*“[The new grocery model] is amazing… People love it. They love being able to take their time. They love having shopping carts, rather than having to carry a box.”*

More appealing and organized displays were seen as effective in encouraging customers to select healthier items and preserving the quality of foods since customers did not need to search through bins of food to find items. Promoting fresh produce through attractive, well-organized, and abundant displays; offering produce bags; allowing customers to self-select produce; and removing restrictions on the amount of produce customers could take were described as particularly impactful.

*“I was calling [a pig farmer to pick up compost] three times a week because all of the produce was being ruined by storing it in crates [and] everyone was digging through it. … So being able to display it in bins inside, and sort it by type, so that it was attractively available, and kind of neat and clean, and whatever. Then my pig farmer only needed to come one day a week because… it was going to families.”*

Other strategies that pantry directors described as feasible and successful included making more space for healthy foods, offering healthy foods that were typically available but infrequently selected (e.g., dry lentils) in multiple locations, and use of “Rinse to Reduce” materials.

### 3.4. Policy Adoption Outcomes

The second intervention component for which the intervention provided technical assistance included the adoption of a food pantry nutrition policy. The food bank housing one pantry adopted an agency-specific nutrition policy. The other 12 pantries collectively developed and adopted a policy for their coalition to “unite staff and volunteers around a clear set of common principles that promote health and nutrition” (See Supplementary Figure S1). Directors completed a worksheet to identify how they intended to prioritize implementation of the coalition policy at their individual pantries. The most frequently reported policy implementation priorities included: emphasizing healthy foods in donor messaging (n = 9), offering a variety of foods (n = 7) and a minimum allotment of fruits and vegetables (n = 7), and designating funds to purchase healthy foods (n = 7). All pantries reported that they intended to implement behavioral economics strategies going forward as outlined in the coalition policy. Three pantries intended to develop agency-specific nutrition standards.

In interviews, directors generally expressed pride and satisfaction with the coalition policy. They indicated that the development process led them to think more about nutrition policies and how a policy could be used to address the unique customers and needs of each pantry than they had previously. Directors described having used or intending to use the coalition policy as a template for or verbatim as their own agency’s nutrition policy and as a communication tool for desired food donations.

*“The conversation …was broadened throughout the experience to really talk about nutrition as a standard of serving with dignity.”*

*“I think that that will put pressure [on food bank distributors] to provide foods that these coalitions have said they want.”*

Some interviewees also discussed limitations to the coalition policy, including the possibility that their organization would not use all components of the policy or that allegiance to the coalition policy might diminish as directors turn over.

### 3.5. Perceptions of the Intervention Approach

#### 3.5.1. Rationales for Participating

Food pantry directors described participating in HFE because it aligned with their organization’s mission and direction. Several participated because they heard how easy or successful it had been for peer pantries. When describing this project to staff and volunteers, most directors did not reference sodium reduction as the primary goal. Instead, they talked about making it easier for customers to choose their own food. Directors emphasized opportunities to promote fresh produce and readily available lower sodium foods often not selected. Some directors also described HFE as an opportunity to reorganize their pantry or make operations run more smoothly.

*“We’ve talked a lot about… healthy foods and how we can make it more accessible for people to read the labels easier, see what they’re picking….”*

*“My key word on this was agency. Giving my clients more freedom to select their own food.”*

#### 3.5.2. How HFE Supported Pantries

Directors said that technical assistance kept the work manageable, provided fresh ideas, and supplied information about behavioral economics.

*“Some of the ideas that [the technical assistance provider] came up with… I see this space every single day, and it just didn’t occur to me.”*

*“If I didn’t have [the technical assistance provider] doing a lot of it, I mean three-quarters of the stuff wouldn’t get done. There’s too many other fires here, day-to-day.”*

*“[The] presentation was really helpful for me to be able to explain to my volunteers and to my staff why I want to set up the distribution area in the way that it is set up.”*

Directors also emphasized that HFE could support pantries ranging in size and styles of service, but readiness for change is important.

*“We were open and available, wanting to hear ideas. I would think if a [pantry] really loves the way that they do things, that that might be kind of difficult.”*

#### 3.5.3. Challenges

Directors described several key challenges experienced while implementing changes based on intervention support, as well as related lessons learned. See Table 4. Directors described facility and space constraints most frequently; followed by challenges related to specific environmental change strategies; and the need for staff, volunteers, and customers to adjust to changes underway. Directors also described challenges related to the availability, quality, and popularity of healthy foods. With regard to nutrition policy development, the overwhelming challenge related to fear of losing donors if pantries refused foods based on nutritional quality.

## 4. Discussion

This study assessed lessons learned from the first two years of a sodium reduction intervention in a diverse group of food pantries. At baseline, more than two-thirds of food items assessed in most categories met standards for consuming “often.” Mixed dishes had the highest sodium content, followed by bread, canned tomatoes and vegetables, and animal/fish-based protein. With intervention support, pantries implemented a range of changes to promote available healthy, lower sodium foods, especially strategies to improve the convenience and visibility of fresh produce. Many also changed their layouts and service models to promote customer choice and dignity. Participating pantries also developed a coalition-level nutrition policy outlining broad commitments and reported plans to implement the policy. Pantry directors reported valuing the intervention approach for its alignment with their organizational missions, flexibility with regard to diverse pantry contexts, and the outside perspective it offered; they also described several key challenges and noted that readiness for change is critical.

Results from the sodium assessments add to the limited body of evidence about the sodium content of pantry foods. Findings indicate that foods with the highest levels of sodium per serving in food pantries align with significant sources of sodium in US diets generally [35]. Pantries in our sample offered a greater proportion of relatively lowsodium options than some might expect given that food pantries are often associated with highly processed, shelf-stable foods. It is important to note, however, that foods lowest in sodium may still be distributed in small quantities [36]. Additionally, the *Guidelines’* sodium standards allow for higher levels of sodium in its optimal tier than is required for foods, such as vegetables, tomatoes, and beans to be labeled “low sodium” per the US Food and Drug Administration (230 mg/serving rather than 140 mg/serving) [34].

Pantry directors’ perceptions of the intervention approach and their descriptions of initial outcomes add to the research base by addressing the acceptability and feasibility of behavioral economics strategies in food pantries [25,26,27]. While other studies have highlighted the effectiveness of behavioral economics strategies, such as bundling and priming in food pantries, participants in this intervention were more likely to implement strategies related to visibility and convenience. Additionally, directors described the value of these strategies as not only nudging customers to select targeted foods but as contributing to a more pleasant and convenient shopping experience for customers—particularly in combination with changes to pantry layouts and opportunities for customer choice. Directors’ decisions about which changes to make centered strongly on the changes’ perceived potential to improve customers’ experiences and increase opportunities for customers to identify and select foods that meet their needs and preferences. This orientation is responsive to previously reported barriers to healthy food access in pantries and indicative of a trend of pantries adopting more holistic approaches including customer-choice models [13,37,38,39]. While the relationship between customer-choice models and the healthfulness of pantry foods is not clear [40], our findings indicate that pantries see customer agency as vital to health promotion and a necessary precursor to nutrition-focused goals, such as sodium reduction. Behavioral economics strategies have been shown to be effective in pantries, though these principles may be less straightforward in pantries than retail settings due to factors that influence decision-making, such as stigma, stress, and restricted choice [26,27,31]. The emphasis on produce among our sample is also notable since produce is both valued by customers and easy for staff and volunteers to recognize as low in sodium without consulting nutrition labels.

While some research exists on policy development among food banks, the literature on policy adoption among food pantries is sparse. One study found that 21% of US pantries have a formal nutrition policy and those pantries reported fewer barriers to offering healthy foods [41]. Other research identified characteristics associated with policy adoption, such as pantry size and the proportion of food purchased [42]. More research is needed to understand the barriers to and facilitators of pantry policy development, as well as policy language that is sufficiently strong to impact food quality yet feasible given the constraints pantries face [42,43]. In this study, participants identified concerns like those identified by food banks, especially fears about damaging donor relationships [44]. One nationwide survey found that nutrition policies do not negatively impact donations for food banks [45]. Still, these concerns may warrant strategies that effectively communicate pantry needs and values to donors to change donation norms.

This study provides insight into how an environmental and policy change intervention can inform practice in a food pantry setting. The assessment approach is one that other food pantries and stakeholders, even with minimal resources, might find useful and feasible. We also provide data on the sodium content of foods where little currently exists and apply a way to interpret those data that could inform others’ food pantry nutrition policy development. To our knowledge, results from the sodium assessments presented here are among the first to use the *Guidelines* to describe the nutritional status of foods in pantry settings. Despite the relatively small sample of participating pantries, the study sample was diverse with regard to key pantry characteristics, such as size and customer demographics, indicating that insights could be relevant to a wide variety of pantries.

The study also has limitations. Pantries were not randomly selected and may differ in important ways from other pantries. We did not examine the potential for differences by pantry characteristics (e.g., pantry size) given the relatively small sample. Sodium assessments were conducted only once per pantry and do not account for potential differences in amounts and types of inventory based on seasonality and other factors. Food items with missing nutritional data or those not assessed may differ in important ways from those that were included in analyses; however, we aimed to address these concerns with the structure of our food categories (e.g., the median sodium content of dry beans is not likely to change substantially by missing data given the generally low sodium content of dry beans). We also refrain from making statements about pantry foods’ overall sodium level since fresh produce items were not assessed and would likely have reduced this estimate considerably. Assessments may not have detected some high sodium contributors since serving sizes are often smaller than normally consumed amounts of some products. This study did not account for the amounts of foods distributed or objectively assess changes in customer food selection. Additionally, not all participating pantry directors were interviewed; some pantries not interviewed were slower to engage in the intervention and made fewer substantial changes. Finally, interviews were conducted by members of the HFE team, so may reflect social desirability bias.

## 5. Conclusions

In conclusion, we found that the intervention approach used was viewed as acceptable and feasible, particularly when explicitly addressing the opportunity for client choice. It shows potential for supporting pantries in improving access to healthy, lower sodium foods and healthier food environments in a manner that accounts for unique pantry contexts and customer agency. This study also found that more than half of foods in 11 food categories had sodium levels in the optimal tier—that is, sodium levels are low enough that the foods could be consumed often. Yet, we also identified food categories that pantries could target for sodium reduction through procurement changes. In combination with evidence that pantry customers often experience difficulty finding foods they need and prefer, our findings reinforce the importance of ensuring that pantries make available healthy options appealing and accessible. Additional research to inform effective strategies for pantry-level nutrition policy development is needed.

## Figures and Tables

**Table 1:** Characteristics of food pantries participating in the Healthy Food Environments intervention in King County, Washington, 2017–2018 (*n* = 13).

Characteristic	Median	Range	*n*
Number of customers served per week	194	112–4000	12
Number of days open per week	3	1–4.25	13
Number of hours open per week	9	3–33	13
Percent of food purchased	10%	0–20%	9
Number of paid full-time staff	2.5	0–4	8
Number of paid part-time staff	1.5	0–5	8
Number of volunteers per week	44	14–95	8

**Table 2:** Number of food items assessed for sodium content by food category, number of items missing data, range and median sodium content of items (mg/serving), corresponding sodium standard threshold for median sodium level, and number of pantries with items assessed in each of 13 food pantries in King County, Washington 2017–2018.

Food Category	Items, *n*	Items Missing Data, n (%)	Sodium (mg) per Serving, Range	Sodium (mg) per Serving, Median	Nutrition Standard Threshold Corresponding to Median Sodium per Serving ^a^	Pantries with Items Assessed, *n*
Beverages, non-milk	579	9 (1.6)	0–920	10.0	Choose sometimes	11
Condiments and sauces	1436	51 (3.6)	0–1210	100.0	n/a ^b^	13
Cooking staples	498	152 (30.5)	0–920	22.5	n/a ^b^	10
Dairy	1509	5 (0.3)	10–450	110.0	Choose often	10
Desserts	1450	244 (16.8)	0–1853	80.0	n/a ^c^	11
Fruit—processed	2093	58 (2.8)	0–150	5.0	Choose often	12
Grains—bread, rolls, tortillas	1162	366 (31.5)	65–1760	290.0	Choose sometimes	11
Grains—cereal	375	42 (11.2)	0–280	135.0	Choose often	10
Grains—other	1801	232 (12.9)	0–1800	0	Choose often	12
Mixed dishes	3319	118 (3.6)	25–1670	610.0	Choose rarely	13
Non-dairy alternatives	190	45 (23.7)	0–180	110.0	Choose often	8
Protein—animal/fish-based	1891	84 (4.4)	25–900	180.0	Choose often	13
Protein—canned beans	1309	2 (0.2)	10–900	140.0	Choose often	13
Protein—dry beans, lentils	1180	730 (61.7)	0–90	5.0	Choose often	10
Protein—nuts, nut butter, tofu	495	4 (0.8)	0–1140	140.0	Choose often	10
Snacks	1210	180 (14.9)	0–990	105.0	Choose sometimes ^d^	11
Tomatoes—processed	1359	3 (0.2)	10–490	220.0	Choose often	13
Vegetables—processed	3595	20 (0.6)	0–590	190.0	Choose often	13

^a^ Nutrition guidelines outline standards for saturated fat, sugar, and in some cases, whole grain ingredients, in addition to sodium for each tier [34]. Here, we only account for the sodium standards corresponding to three tiers: choose often, choose sometimes, or choose rarely. ^b^ Nutrition standards for this food category do not specify values for the “choose often,” “choose sometimes,” or “choose rarely” tiers. ^c^ Nutrition standards for this food category classify all foods as “choose rarely”. ^d^ Based on the nutrition standards for this food category, foods could only be classified as “choose sometimes” or “choose rarely”.

**Table 3:** Number of food pantries that implemented each of 11 environmental change strategies in King County, Washington 2017–2018 (*n* = 13).

Environmental Change	Number of Pantries	Change Details
Behavioral economics strategies		
Improve visibility or salience	11	Replaced food crates with shelving, bread racks, and tilting produce stands Called attention to produce with attractive awnings, baskets, or bins Placed healthiest food options at eye level Installed new lighting over healthy foods Created the appearance of plentiful produce by filling in the base of produce bins with other materials Used labels and glass panels to identify items stored behind freezer and cooler doors
Improve convenience	11	Moved foods from crates to shelving to minimize the need to bend over or sift through items Used tilting produce stands, bins, and well-spaced shelving so healthy food options are within easy reach If foods were previously distributed outside, moved them indoors Offered produce bags to aid customers in carrying fruits and vegetable
Placement, ordering, and priming	6	Displayed healthy foods in central locations Offered healthy foods at multiple points in the distribution line or shopping path, including once early in the line and again near the end to maximize selection opportunities Created more space for healthy foods relative to unhealthy foods
Signage	5	Used banners and posters to highlight fresh produce Used pictorial signs to label freezer sections so multilingual customers and customers with dietary restrictions could identify what they needed or wanted to avoid Posted “Rinse to Reduce” posters, shelf labels, and can toppers near canned beans and vegetables to promote sodium reduction
Recipes	3	Posted recipe racks to suggest cooking ideas for pantry foods
Cooking demonstrations	3	Offered or trained volunteers to offer cooking demonstrations highlighting healthy options
Nutrition labeling	0	Provided information about the nutritional quality of foods
Normalizing	0	Conveyed positive norms about consumption of food items
Bundling	0	Bundled foods together that are complementary or can make a meal
Other environmental changes		
Offer more customer choice or experiences similar to a grocery store	8	Transitioned from a line of service to a customer-directed format Transitioned from pre-boxed foods to customer self-selection Offered grocery carts, check out stations, and produce bags to customers
Changes to pantry layout for improved customer experience	7	Created lobbies and more spacious pathways for customers Created line efficiencies so customers experienced less waiting time

**Table 4:** Challenges to implementing environmental and policy changes as described by food pantry directors in King County, Washington 2017–2018 (*n* = 10).

	Challenge	Illustrative Quotes	Lessons Learned
Facility and space constraints	Many pantries operated out of older, relatively small, rented spaces not designed for food distribution. This presented difficulties in allowing for ideal pathways, lighting, or display structures.	*“It’s hard to create a welcoming, inviting space … in an old warehouse, gym space.”*	This challenge led some pantries to make adjustments to their layouts and shopping pathways to improve customer flow at the same time they worked on applying behavioral economics principles to highlight individual foods. Pantries also opted to purchase equipment to address these challenges, including additional lighting and items on wheels that could be moved as needed.
Strategy-specific challenges.	Some challenges were specific to a change or strategy being implemented. For example, signage was difficult to maintain or minimally effective in some cases due to space constraints and multilingual needs. Presenting healthy foods first in distribution lines conflicted with typical practices of offering produce last so it would not be crushed by heavier items in carts or bags. Conducting food demonstrations required volunteers and space not always available.	*“We did try having a lot of signage… trying to direct people to healthy choices, and we didn’t find it very effective. It was almost more in the way. We found that the more signs that were posted, the less people read them.”*	Technical assistance providers encouraged pantries to experiment with and adapt changes without feeling they had to commit to any change permanently.
Adjusting to change.	Many directors described initial difficulties associated with some staff, volunteers, and customers resisting changes made. This was especially true for individuals who had long-term history with the agency and were accustomed to things being done in a particular way.	*“The people who are still griping—it’s about change. It’s not really about the system.”*	All directors who described this challenge said it was typically a short-lived concern. Many also stressed the importance of involving staff and volunteers in the planning process along the way to support buy in for the changes.
Availability, quality, and popularity of healthy foods.	Some directors described ways in which their best efforts to create healthy food environments were limited by the foods available to them and indicated this was often beyond their control. For example, pantries typically set allotment restrictions to ensure that most customers get some of the most desirable foods. This inherently limits customer choice. One director said it would be helpful to focus policy development further upstream in the food supply.	*“I don’t like to put limits on food. But then on the other hand, I don’t like to run out of food.”* *“It should’ve been implemented from the grocery store’s perspective, from [the food bank distributor’s] perspective, USDA’s perspective… So that when we get government commodities, I don’t get regular canned corn; I would get low-sodium corn.”*	Several directors noted that, though they feared additional customer choice might result in them running out of popular items, this often did not occur as customers typically only took the amount they needed and wanted to leave some for others. Additionally, the intervention envisioned that concurrent work on nutrition policy development and implementation would lead to a healthier food supply.
Fear of losing donors based on nutritional quality standards instituted by nutrition policies.	Some directors expressed concern or resistance to policy commitments outlining foods to be refused based on nutrition standards because they worried that the donor would then choose not to provide any food. A smaller number of directors expressed concerns about not wanting to “police” the food that should be provided to pantry customers.	*“How do we say no to a donation? … That’s really hard for some people who are in this work.”* *“If we get to the point where we create absolute lists on what we want and what we don’t want, I feel like there’s a lot of those people that donate to us are going to say, ‘Well, forget it then. I’ll find somebody that will take it all.’”*	To address this concern, technical assistance providers began inviting pantries experienced in implementing nutrition policies to present at nutrition policy development training. These invited guests were specifically asked to address whether they had lost donors after implementing nutrition standards for donated food; most indicated they had not. Pantries were also encouraged to orient their nutrition policies toward identifying foods they would prioritize for purchase or request from donors as a first step, before identifying foods they would refuse if this was a concern.

## Data Availability

The data presented in this study may be available on request from the corresponding author. The data are not publicly available as permission for this was not sought during the informed consent and data collection process.

## Supplementary Materials

The following are available online at https://www.mdpi.com/article/10.3390/ijerph182413206/s1, Figure S1: Policy commitments from the *South King County Food Coalition Nutrition Standards*, 2018.
