# Supplementary material for: Healthy Food Environments in Food Pantries: Lessons Learned from a Sodium Reduction Intervention
Source: Int J Environ Res Public Health. 2021 Dec 15;18(24):13206. doi: 10.3390/ijerph182413206 (PMC8701667; doi:10.3390/ijerph182413206)
Supplement: Supplementary file 1 [file ijerph-18-13206-s001.zip › ijerph-1471863-supplementary.pdf]

1) We will serve our clients with **dignity**.
   - We will create environments that allow clients to have more freedom to choose which foods fit their dietary needs.
   - We will provide healthy choices.
   - We will serve with abundance.

2) We will put our clients' health first by prioritizing the distribution of recommended **USDA's MyPlate** foods such as:
   - Fruits and vegetables, especially fresh
   - Whole grains
   - Low-fat or non-fat dairy and milk products
   - A variety of protein foods
   - Foods lower in sodium and with less added sugar

3) We will provide **more fresh foods**.
   - We will seek to supplement donated foods with the purchase of fresh foods as well as distribution of Elk Run Farm produce.
   - When purchasing food, we will seek to limit processed foods to those that are low in sodium and added sugars.

4) We will use **behavioral economics** strategies to highlight healthy food options so the healthy choice is the easy choice.

5) We will provide **nutrition education** about the foods that we distribute and information on how to prepare them.
   - We will focus education on key health concerns for our region, especially high blood pressure and diabetes.
   - We will provide information like recipes and pamphlets
   - When possible, we will translate materials into languages other than English.
   - We will provide food samples and cooking demonstrations when possible.

6) We will **educate our community** about the disparities that exist in South King County.
   - We will inform our donors about the importance of donating foods that help to improve our clients' health.
   - We will highlight to our community members the importance of their neighbors' health and provide them with a forum to enact change.

Figure S1. Policy commitments from the *South King County Food Coalition Nutrition Standards*, 2018.
